# Induction of Biofilm Formation in *Klebsiella pneumoniae* ATCC 13884 by Several Drugs: The Possible Role of *Quorum Sensing* Modulation

**DOI:** 10.3390/antibiotics7040103

**Published:** 2018-11-28

**Authors:** Elizabeth Cadavid, Sara M. Robledo, Wiston Quiñones, Fernando Echeverri

**Affiliations:** 1 Grupo de Química Orgánica de Productos Naturales, Instituto de Química, Universidad de Antioquia, Calle 67 No. 53-10, Medellín 050010, Colombia; elicatoqf@gmail.com (E.C.); wiston.quinones@udea.edu.co (W.Q.); 2 PECET, Instituto de Investigaciones Médicas, Facultad de Medicina, Universidad de Antioquia, Calle 62 No. 52-59, Lab 632, Medellín 050010, Colombia; sara.robledo@udea.edu.co

**Keywords:** *Klebsiella pneumoniae* ATCC 13884, virulence, resistance, *quorum sensing*, drugs, biofilm, acetaminophen, hydrochlorothiazide

## Abstract

Bacterial resistance is caused by several biochemical factors, the formation of biofilm being one of the main causes. This process is triggered by *Quorum Sensing* (*QS*), through the production of endogenous molecules, although other substances such as natural products can also do this. In this work, we aimed to determine whether some drugs are involved in the induction of biofilm formation in *Klebsiella pneumoniae* ATCC 13884, and thus, increase bacterial resistance. For this, the effect of 22 drugs on *K. pneumoniae* ATCC 13884 growth was determined at sub-plasmatic concentrations; the production of autoinducer lactones was established by HPLC and with a biosensor. The induction of biofilm formation was determined through crystal violet assay at 585 nm in a microplate reader and using urethral catheters. According to the in vitro assays, some drugs were found to induce biofilm formation in *K. pneumoniae* ATCC 13884. The effect of acetaminophen, hydrochlorothiazide, and progesterone stood out. The first drug caused several changes in the biochemistry of *K. pneumoniae* ATCC 13884 related to *QS*: high synthesis of *N*-hexanoyl-homoserine lactone, increasing bacterial populations by 27% and biofilm formation by 49%, and a more gentamicin resistant biofilm. Furthermore, it increased the colonization area of urethral catheters. Hydrochlorothiazide showed the biggest increase in the induction of biofilm formation of 51%, and progesterone displayed the greatest ability to provoke bacterial mass adherence but had no effects on *K. pneumoniae* ATCC 13884 bacterial population growth.

## 1. Introduction

Antibiotic resistance has become a threat to humanity causing hundreds of thousands of deaths annually and dramatically increasing treatment time and its costs. *Klebsiella pneumoniae* is the causal agent of several diseases including pneumonia, meningitis, bloodstream infections, and surgical site infections. Furthermore, some strains are resistant to carbapenem antibiotics, including gentamicin and ciprofloxacin [1], and are the most commonly isolated bacterium from medical devices [2,3]. The World Health Organization classified *K. pneumoniae* as a critical priority and established the need to promote the search for new antimicrobial agents [4].

*K. pneumoniae* and other bacteria exhibit *Quorum Sensing* (*QS*), a mechanism of bacterial communication, which regulates some biochemical processes in planktonic cells, and biofilms [5]. *QS* is involved in pathogenicity and virulence through modifications in motility, the synthesis of hydrolytic enzymes, toxins, and polysaccharides, as well as biofilm formation [6], among other things. Biofilm formation represents a major resistance mechanism, since it renders bacterial colonies impermeable to antibiotics [7], promotes the transmission of resistance genes, increases the expression of efflux pumps, increases antibiotic metabolism, and favors persister cells [8]. 

Although increases in recurrent infections have been attributed to processes inherent to pathogen biochemical resistance, QS offers a better understanding of the factors that induce biofilm formation. Therefore, it is essential to determine whether exogenous molecules can induce QS in *K. pneumoniae.* For this reason, the present study aimed to determine whether some drugs used in the treatment of several diseases could be involved in the formation of *K. pneumoniae* ATCC 13884 biofilm, and, thereby, increase bacterial resistance. The understanding of this mechanism is likely to result in improved clinical procedures, such as an optimal pharmacologic treatment [9] that avoids exposing patients to these drugs and thus prevents recurrent infections.

This work was conducted through the following steps: (1) The effects of drugs on bacterial growth and biofilm formation were determined. Due to its importance as an OTC drug, the next assays were made only with acetaminophen. (2) The presence of C6AHL autoinducer was established and quantified. (3) The effect of acetaminophen on mature biofilm was analyzed. (4) The architecture of biofilm was observed by microscopy. (5) The effect on the adherence to urethral catheters was also determined.

## 2. Materials and Methods

### 2.1. General

#### 2.1.1. Drug Selection

Twenty-two drugs were selected from a list of essential medications and were kindly donated by Laboratorios LAPROFF (Sabaneta, Colombia). The drugs were taken from a list of essential drugs and based on their frequency of use. These drugs are mainly used for chronic diseases, or as antibiotics. Later, progesterone and cholic acid were added.

*N*-Acyl-homoserine lactone autoinducer was obtained from Sigma-Aldrich (St. Louis, MO, USA).

#### 2.1.2. Bacteria

*Chromobacterium violaceum* CV026 mutant was donated from Dr. Walter Giordano from the University of Rio Cuarto (Argentina). *K. pneumoniae* subsp. *rhinoscleromatis* ATCC 13884 was purchased from American Type Cell Collection (Manassas, VI, USA). These were cultured in Luria–Bertani Agar (LB) (Lab M, Lancashire, UK) and incubated at 37 °C (*K. pneumoniae*) or 28 °C (CV026). For all experiments, the inoculums were prepared in saline solution 0.9%; assays were performed in triplicate unless otherwise stated. The absorbance of violacein was determined at 585 nm in a microplate reader (Thermo Scientific, Waltham, MA, USA).

#### 2.1.3. Equipment

HPLC (Agilent 1200 series system, Santa Clara, CA, USA) coupled with a Quadrupole Mass Spectrometer (VL model) was used and operated in the Electrospray Ionization (ESI) mode, with the Agilent ChemStation 04.03.087 software, equipped with a 4.6 mm × 150 mm, 5 µm ZORBAX Eclipse XDB-C18 analytical column. Analysis was carried out at a temperature of 35 °C with a flow rate of 0.8 mL/min, and an injection volume of 5 µL. Water with 0.1% formic acid and acetonitrile with 0.1% formic acid were used as mobile Phases A and B, respectively. Solvent gradient B from 60% to 90% was used for a run time of 8 min.

Urinary catheter from PVC Medex^®^ (Passaic, NJ, USA) caliber N° 20 was used.

### 2.2. Effect of Drugs on *K. pneumoniae* ATCC 13884 Growth

The effects of drugs on *K. pneumoniae* growth were determined using the microdilution method, as described by Wiegand [10], but using 100 µL of inoculum, equivalent to 10^6^ CFU/mL.

### 2.3. Effect of Drugs on *K. pneumoniae* ATCC 13884 Biofilm Formation

#### 2.3.1. Kinetics of Biofilm Formation

The maximum incubation time to obtain a greater bacterial mass was determined using 96-well microplates according to O’Toole [11], but using LB medium and incubating for 30 h, using crystal violet (0.05% in water). 

#### 2.3.2. Biofilm Formation

The inoculum was prepared from a colony grown on LB agar for 24 h, in a 0.9% saline solution and adjusted to OD_600nm_ 0.05; then, 100 µL was added to each well with 100 µL of LB medium containing drugs at non-biocide plasmatic concentrations. The microplates were incubated at 37 °C for 30 h. The attached bacterial mass measurement was carried out as previously stated. The amount of biofilm was calculated by comparison with the growth of untreated bacteria.

### 2.4. Production of N-Hexanoyl-Homoserine Lactone, C6-AHL

#### 2.4.1. Detection Using the Biosensor *C. violaceum* CV026

A violacein absorbance calibration curve was made to quantify the C6-AHL concentration. C6-AHL, at concentrations between 0.06 and 100 µg/mL, was added to 100 µL of sterile *K. pneumoniae* ATCC 13884 supernatant, 50 µL of LB medium, and 50 µL of *C. violaceum* CV026 inoculum with DO_600nm_ of 0.1. The final concentrations of C6-AHL were 0.03, 0.05, 0.10, 0.20, 0.39, 0.78, 1.56, 3.12, 6.25, 12.5, 25, and 50 µg/mL. This mixture was incubated at 28 °C for 48 h and dried at 50 °C for 12 h. The violacein was extracted with 250 µL of methanol and the absorbance was determined at 585 nm. To establish the relationship between violacein absorbance and concentration of C6-AHL, a simple linear regression model was used, determining the correlation coefficient and the slope.

#### 2.4.2. Detection of C6-AHL by HPLC/MS

A culture of 250 mL of *K. pneumoniae* ATCC 13884 in an LB medium previously adjusted to OD_600nm_ of 0.05 was incubated for 2, 4, 6, 8, and 23 h. At the end of each period, each sample was sonicated for 5 min and centrifuged at RCF = 1132 for 10 min and the supernatant was acidified at pH 3.5 with 10% acetic acid, and then lyophilized. The dried material (2 g) was extracted with 80 mL of ethyl acetate. Then, the solvent was vacuum-dried, and the material was re-dissolved in 2 mL of methanol. HPLC analysis was performed as described by Yin [12]. *N*-hexanoyl-homoserine lactone standard was employed to compare the retention times and a fragment at *m*/*z* 200.24 was monitored.

#### 2.4.3. Kinetics of C6-AHL Production

An inoculum of *K. pneumoniae* ATCC 13884 was prepared in saline solution with an OD_600nm_ of 0.05 ± 0.01. LB medium was added at a 1:1 ratio (*v*/*v*) and the mixture incubated at 37 °C for 24 h; supernatant aliquots were collected at 2, 4, 6, 8, and 23 h. Next, samples were filtered through 0.2 µm pores. From the filtered supernatant, 100 µL were added to 50 µL of *C. violaceum* CV026 inoculum adjusted to OD_600nm_ 0.05 ± 0.01 and 50 µL of sterile LB medium. The concentration of C6-AHL in the supernatant was lower than the biosensor detection limit, so all samples were doped with 0.2 µg/mL of C6-AHL to establish the minimum concentration of lactone needed to induce violacein production. After incubating the *C. violaceum* CV026 biosensor at 28 °C for 48 h, the microplate was dried at 50 °C for 24 h. The dried material was then extracted with 250 µL of methanol and absorbance was determined at 585 nm.

#### 2.4.4. Production of C6-AHL in *K. pneumoniae* ATCC 13884 after Drug Treatment

The levels of AHLs in the *K. pneumoniae* ATCC 13884 supernatants after treatment with biofilm-inducing drugs were detected by assessing the levels of violacein synthesis by *C. violaceum* (CV026). However, to do that, it was first necessary to establish the drugs’ ability to activate QS in these bacteria. Therefore, 100 µL of inoculum, adjusted to OD_600nm_ 0.05, was added to 96-well microplates containing 100 µL of LB medium, with each drug at a final concentration of 1 µg/mL. Samples were incubated for 48 h, then violacein was extracted with 250 µL of methanol and finally absorbance was determined at 585 nm. 

Drugs with a high biofilm-inducing effect were added to the *K. pneumoniae* ATCC 13884 cultures in LB media at the same concentrations exhibited for induction. Samples were incubated at 37 °C for 3 h, the supernatant was filtered through 0.2 µm pores and then evaluated with the *C. violaceum* CV026, as above. A drug-free culture supernatant containing 0.2 µg/mL of C6-AHL (doping) was used as control.

### 2.5. Effect of Drugs on the Resistance of *K. pneumoniae* ATCC 13884 Mature Biofilms

#### 2.5.1. Minimum Antibiotic Concentration Required to Eliminate Mature Biofilms

To establish the stability of the biofilms, the antibiotics gentamicin and ciprofloxacin were chosen. The first one was selected because *K. pneumoniae* ATCC 13884 shows resistance to it [13], and the latter because of its ability to cross the biofilm wall [14] and act upon bacteria embedded there.

A culture of *K. pneumoniae* ATCC 13884 was incubated for the maximum biofilm formation time, as described above in Section 2.3.1. *Kinetics of Biofilm Formation*. After the formation of a mature biofilm, the supernatant was removed, and the sample was rinsed with sterile water. A mix of LB medium in 50% saline solution with ciprofloxacin and gentamicin was again added to each well at concentrations of 0.0015–1.0 µg/mL. The plates were incubated at 37 °C for a further 18 h and then washed twice with sterile water. Each well was treated with 0.05% crystal violet to quantify the remaining bacteria and to establish the antibiotic concentration enabling to remove 50% of *K. pneumoniae* biofilm.

#### 2.5.2. Mature Biofilms Formed under Biofilm-Inducing Drugs

*K. pneumoniae* ATCC 13884 culture was incubated in the presence of acetaminophen, hydrochlorothiazide, and progesterone for the maximum biofilm formation time. The microplates were rinsed twice with distilled water. Then, 200 µL of gentamicin (concentration 0.1 µg/mL in LB medium) were added. After that, they were incubated at 37 °C for 18 h, and then the supernatant was removed, and each well was rinsed with sterile water and dried at 50 °C. Finally, the samples were treated with 0.05% crystal violet solution to establish the remaining amount of mature biofilm. 

#### 2.5.3. Architecture of the Mature Biofilm

Liquid LB medium containing biofilm-inducing drugs and *K. pneumoniae* ATCC 13884 inoculum in saline solution at OD_600nm_ 0.05 was placed on 1 cm × 1 cm glass coverslips and incubated at 37 °C for 30 h. Afterwards, the coverslips were submerged once in sterile water. For staining, the coverslips were submerged in 0.1% acridine orange in Plasma Bovine Serum (PBS) at pH 7 for 1 min and then the excess dye was rinsed off with sterile water. The stained biofilm was imaged in an Episcopic-Fluorescence Attachment EFD-3, using a B-2A fluorescence filter cube coupled to with camera. 

### 2.6. Effect of Acetaminophen on *K. pneumoniae* ATCC 13884 Adherence to Urethral Catheters

A piece of 5 cm, 20-caliber PVC catheter Medex^®^ (Passaic, NJ, USA) was submerged in LB culture medium with *K. pneumoniae* ATCC 13884 adjusted to OD_600nm_ of 0.05 in saline solution and containing acetaminophen at plasmatic concentrations (Table 1); the same drug-free medium was used as negative control. Cultures were incubated at 37 °C for 30 h and catheters were submerged in sterile water to remove sessile bacteria. Samples were then dried at 50 °C for 2 h. Pictures were taken with an optical microscope and scanning electron microscope. 

For the optic microscope technique, the catheters were submerged in 0.05% crystal violet solution for 5 min and then rinsed with sterile water and dried at 50 °C for 2 h. Afterwards, the catheters were photographed with a binocular microscope using 4× and 10× objectives. Biofilm sizes and their appearances were analyzed by ImageJ program. All experiments were performed in duplicate.

For SEM, after drying, the catheters were covered with nebulized gold and photographed at 200X magnification.

### 2.7. Statistical Analysis

The assays underwent a statistical analysis to compare means using Multiple Range Tests, with a confidence level of 95%, using Statgraphic Centurion software (Version 16.2.04, The Plains, VA, USA).

## 3. Results

To determine the effects on *quorum sensing* mechanisms without interference from the biocide effect, all drugs were analyzed at sub-lethal concentrations; these concentrations were established by finding the concentration range at which bacteria exhibited a growth equal to or higher than that of untreated bacteria. Before measuring the levels of *N*-Acyl-homoserine lactone, the effect of the drugs was established at concentrations of 1.0 µg/mL directly on the CV026 biosensor, without C6-AHL doping. None of them were found to induce violacein synthesis above the minimal detection limit of absorbance (OD 0.05 UA at 585 nm).

The concentrations of active ingredients were adjusted with LB medium to be within the range of plasmatic concentrations, which were obtained from the Drugbank online database, Version 5.0 (https://www.drugbank.ca/); the specific range for each drug is shown in Table 1.

### 3.1. Effect of Drugs on *K. pneumoniae* ATCC 13884 Growth

Gentamicin and tetracycline caused a large increase in the bacterial population, 31% higher than that of the control (Table 2). Amikacin, nitrofurantoin, norfloxacin, levofloxacin, and ciprofloxacin did not affect bacterial growth. Both gentamicin and tetracycline were selected as positive controls for the following experiments.

When drugs were assayed, three groups were noted according to their effects. The first group included cetirizine, loratadine, propranolol, metformin, and lovastatin; these drugs did not modify *K. pneumoniae* ATCC 13884 growth (Table 3). The second group comprised enalapril, captopril, diclofenac, dexamethasone, and progesterone; these drugs caused a slight increase in bacterial populations (up to 15%). The third group of drugs contained acetaminophen, carbamazepine, verapamil, cholic acid, and hydrochlorothiazide; these drugs increased bacterial growth by 16–31%. 

### 3.2. Effect of Drugs on *K. pneumoniae* ATCC 13884 Biofilm Formation

#### 3.2.1. Kinetics of Biofilm Formation

**Figure 1:**
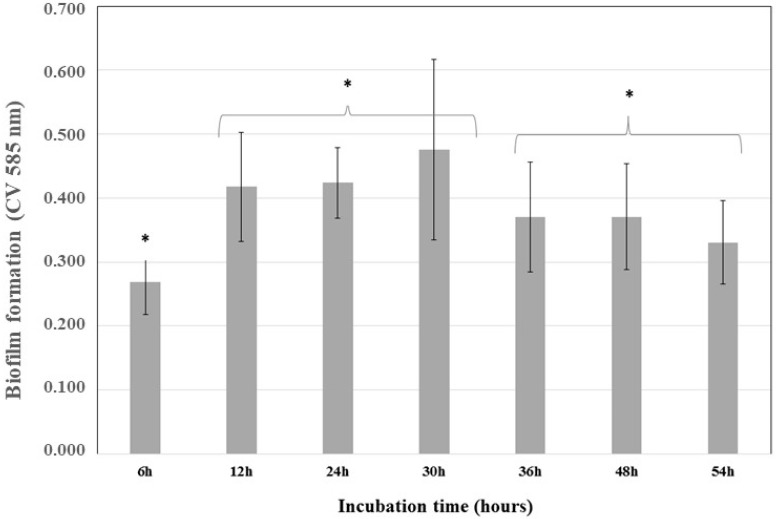
Kinetics of *Klebsiella pneumoniae* ATCC 13884 biofilm formation. *K. pneumoniae* ATCC 13884 was incubated for 54 h and the biofilm was analyzed at 6, 12, 24, 30, 36, 48, and 54 h to determinate the best time for maximum biofilm formation. The highest value was 30 h and indicated the time required to obtain a mature biofilm. * Statistically significant differences were observed for 6 h, 12–30 h, and 36–54 h (* *p* < 0.05) (*n* = 15).

After incubating *K. pneumoniae* ATCC 13884 for 54 h, the absorbance of crystal violet at 585 nm was plotted against time (Figure 1). From these kinetics analyses, the highest bacterial adherence time was 30 h. Afterwards, a reduced adherence was detected, indicating nutrient depletion and biofilm detachment.

#### 3.2.2. Biofilm Formation

The previously mentioned drugs were tested for their ability to induce biofilm formation. Of all 22 drugs assayed, 11 increased biofilm formations (Table 2 and Table 3).

The antibiotics amikacin and norfloxacin were the most powerful inducers of biofilm formation, increasing it by 56% and 50% compared to the control, respectively; however, they did not change the bacterial growth. Tetracycline increased biofilm formation by 46% and increased the bacterial population by 31%. Gentamicin at 0.01 µg/mL also increased biofilm formation by 25% and bacterial population by 31%; these results are comparable to those previously reported for *K. pneumoniae* resistance through the activation of Extended Spectrum Beta Lactamase (ESBL) synthesis and increase in biofilm formation (7). It has been reported that 42.9% of nosocomial isolates of the genus *Klebsiella* are resistant to this antibiotic [15].

Three other drugs, enalapril, diclofenac, and carbamazepine, had no effect on biofilm formation, despite increasing bacterial growth. Loratadine had no effect on either bacterial growth or biofilm formation.

The steroids dexamethasone and cholic acid did not provoke positive effects on biofilm formation since induction was lower than 14%. However, there is a report that states that an addition of 1% of bile salts to the culture induced biofilm formation in *K. pneumoniae* [16]. Surprisingly, progesterone exhibited a strong effect on biofilm formation, increasing it by 65%, surpassing tetracycline (46%) despite showing a lower growth induction of only 11%.

The following drugs also had a great impact on the induction of biofilm formation: hydrochlorothiazide (51%), acetaminophen (49%), and captopril (24%). The first two exceeded the inducing effect of tetracycline and simultaneously showed a positive effect on bacterial growth, 17% and 27%, respectively.

The results demonstrate that some drugs play an active role in biofilm formation in *K. pneumoniae* ATCC 13884. However, there was no positive relationship regarding bacterial growth.

### 3.3. Production of C6-AHL

#### 3.3.1. Detection of C6-AHL through the Biosensor *C. violaceum*

The curve of violacein absorption vs. C6-AHL concentration showed that violacein production was induced to its maximum peak at approximately 3.0 µg/mL and maintained a linear relationship between 0.03 and 0.39 µg/mL. This range was used to obtain the following equation and to establish C6-AHL concentrations in the supernatants of cultures after treatment with each drug:

(1) Violacein abs=0.07761+0.26255∗X µg/ml of C6 AHL

The equation has a correlation coefficient of 0.8850 and a standard error of 0.0187. 

The quantity of C6-AHL necessary to activate violacein synthesis in the biosensor was also determined, since the AHL concentration in *K. pneumoniae* ATCC 13884 supernatants is close to the lower quantification limit of the biosensor. Therefore, the remaining assays with *C. violaceum* CV026 were doped with 0.2 µg/mL of C6-AHL.

#### 3.3.2. Detection by HPLC/MS

To assess the presence of C6-AHL, the supernatant cultures were analyzed by HLPC/MS, using a standard compound and monitoring the 200.24 fragment [M + H]^+^. C6-AHL was detected at a concentration of 0.05 ± 0.009 µg/mL in the control sample. 

#### 3.3.3. Kinetics of C6-AHL Production in *K. pneumoniae* ATCC 13884

**Figure 2:**
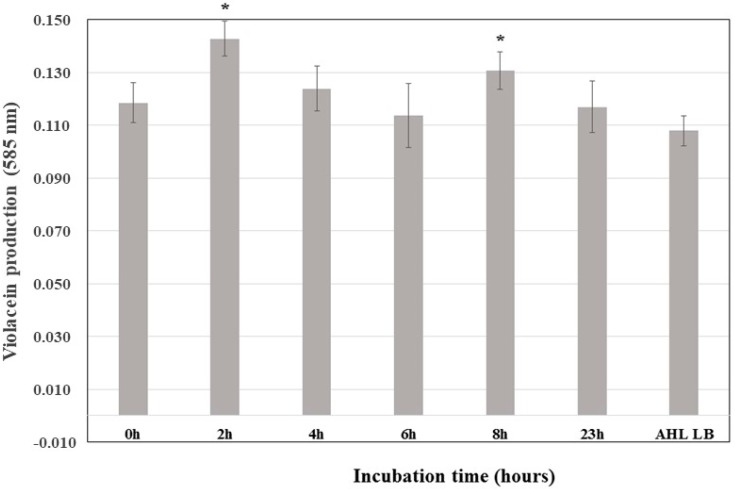
Kinetics of the production of C6-AHL in *Klebsiella pneumoniae* ATCC 13884 cultures. All samples were doped with 0.2 µg/mL C6-AHL; a control experiment was conducted but without *K. pneumoniae* ATCC 13884. * Statistically significative difference.

Violacein synthesis in the *C. violaceum* CV026 biosensor increased significantly after adding the supernatant of *K. pneumoniae* ATCC 13884 incubated for 2 h, revealing that the highest rate of C6-AHL synthesis occurs during the exponential phase (Figure 2). Therefore, analyses of the amount of C6-AHL synthetized in the presence of biofilm-inducing drugs were performed between 2 and 4 h of incubation.

#### 3.3.4. Production of C6-AHL in *K. pneumoniae* ATCC 13884 after Drug Treatments

Supernatants of *K. pneumoniae* culture treated with acetaminophen at concentrations between 0.3 and 2.5 µg/mL increased violacein production by 10%, which is equivalent to C6-AHL concentrations of 0.35 ± 0.02 µg/mL. Meanwhile, control-induced violacein production was 0.26 ± 0.02 µg/mL of C6-AHL; therefore, acetaminophen at plasmatic concentrations increased the production of this compound by 22%. Other drugs, such as hydrochlorothiazide and progesterone, showed no significant effect on the production of this autoinducer (Table 4).

### 3.4. Effect of Drugs on Mature Biofilm Resistance in *K. pneumoniae* ATCC 13884

#### 3.4.1. Minimum Antibiotic Concentration Required to Eliminate Mature Biofilms

**Figure 3:**
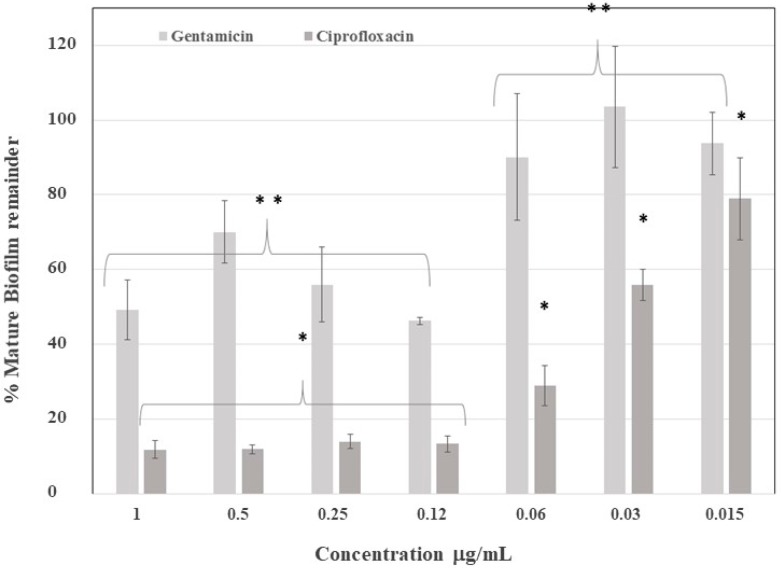
Mature biofilm elimination after application of antibiotics. Gentamicin eliminated 50% of the mature biofilm at between 0.12 and 1.0 µg/mL, while ciprofloxacin eliminated 50% of the mature biofilm at 0.03 µg/mL. Calculated values are in respect to the control group mean. ** Statistically significant difference between groups for gentamicin. * Statistically significant difference between groups for ciprofloxacin.

The mature biofilm was exposed to ciprofloxacin and gentamicin to establish the antibiotic concentration necessary to eliminate 50% of the biofilm. Accordingly, the biofilm was up to four times more resistant to gentamicin, as compared with ciprofloxacin (Figure 3), because gentamicin was only able to eliminate 50% of the biofilm at 0.12 µg /mL, while ciprofloxacin achieved this at 0.03 µg/mL. This difference can be explained by ciprofloxacin’s ability to cross biofilms, thus performing its antibiotic effect on the embedded bacteria.

#### 3.4.2. Effect of Antibiotics on Mature Biofilm Formed by Drugs

**Figure 4:**
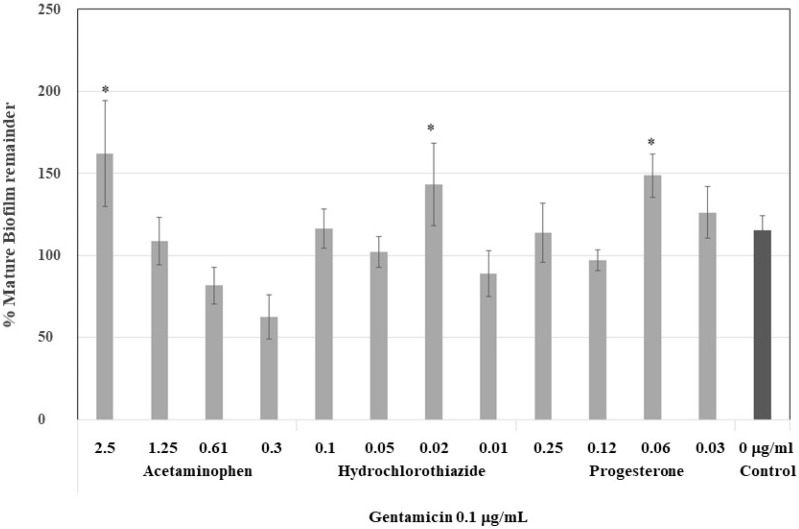
Effect of gentamicin on the stability of the biofilm formed under biofilm-inducing drugs at plasmatic concentrations. Mature biofilm formed after 30 h of drug applications was treated with gentamicin 0.1 μg/mL, and then, the mature biofilm remainder was quantified. In acetaminophen, a drug-dependent decrease was noticed at higher concentrations of 2.5 and 1.25 μg/mL. * Statistically significative difference.

To demonstrate the strength of the induced biofilm, mature biofilms were treated with a dose of gentamicin required to eliminate 50% of the biofilm, that is, 0.12 µg/mL. At higher concentrations of acetaminophen, 2.5 and 1.25 µg/mL, the biofilm was more resistant to gentamicin, nearly 47% of the control (Figure 4). However, the biofilm which formed at low concentrations of acetaminophen was less resistant—even less resistant than those formed at low concentrations of hydrochlorothiazide and progesterone. These drugs produced more stable biofilms which were resistant to gentamicin but at lower concentrations (0.02 and 0.06 µg/mL, respectively).

#### 3.4.3. Effect on Mature Biofilm Architecture

**Figure 5:**
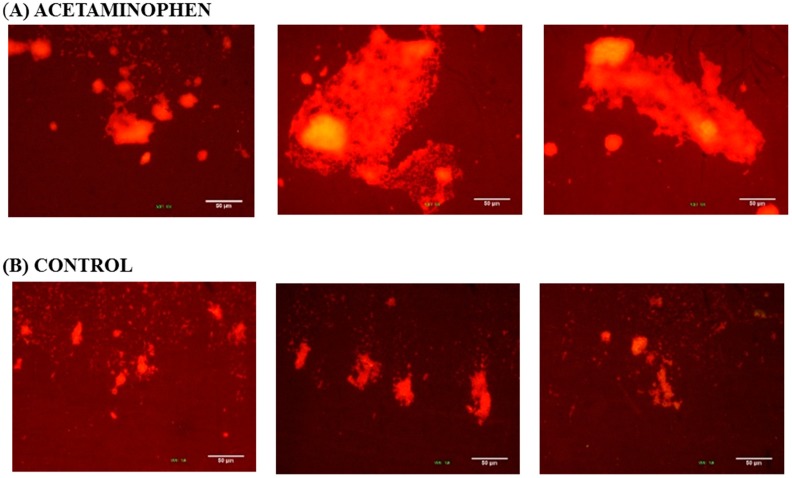
*K. pneumoniae* ATCC 13884 biofilm formed on coverslip glass. The images were taken from three different zones on the coverslip glass. The brighter areas indicate greater accumulation of bacteria. (**A**) Biofilm assembled under acetaminophen has more bacterial aggrupation and a larger colonized area. (**B**) The control group has few bright areas and less bacteria adherence. Bar 50 μm.

Three images at 10× magnification were taken of each biofilm sample formed on coverslips after staining with 0.1% acridine orange. The biofilm size is indicated by the bright area and the population density is indicted by brightness color. In the presence of 0.6 µg/mL acetaminophen (Figure 5A), the *K. pneumoniae* ATCC 13884 biofilms enlarged their colonization area and reduced the inter-bacterial spaces. As result of the increase in population density, a more compacted structure was formed. As can be seen in the control images (Figure 5B), the samples which were not treated formed smaller aggregates with fewer bright areas and flatter configurations were formed.

### 3.5. Effect of the Drugs on *K. pneumoniae* ATCC 13884 Adherence to Urethral Catheters

**Figure 6:**
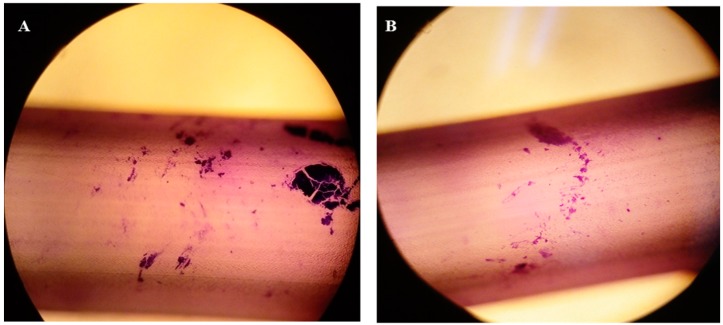
Optical microscope image of the *K. pneumoniae* ATCC 13884 biofilm assembled on the urethral catheter: (**A**) biofilm formation during acetaminophen treatment; and (**B**) without treatment with acetaminophen; both stained with crystal violet at 0.05%. Quantification of the area for each treatment was carried out using the ImageJ program, processing the sample by the particle content. Thus, the colonized area in the control sample was 2.07%, while in the acetaminophen sample it was 12.17%.

Quantification of the colonized area for each treatment was carried out using the ImageJ program. Thus, the colonized area of the bacteria under acetaminophen treatment was 12.17%, while under control conditions it was 2.07%. For both samples, an area of 1,735,237 µm^2^ was evaluated. In the presence of acetaminophen, the bacteria colonized a greater area on the PVC catheters (Figure 6A). In addition, more compact structures were observed in comparison with the biofilm produced without drug treatment (Figure 6B), with more dispersed aggregates.

**Figure 7:**
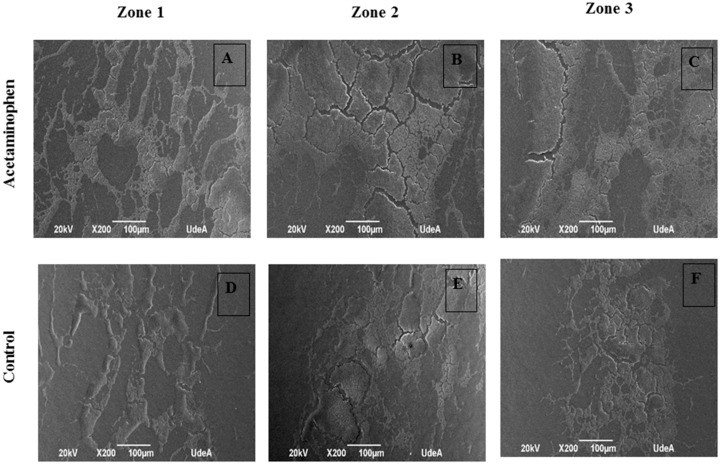
Scanning electron microscope Image of *K. pneumoniae* ATCC 13884 biofilm assembled on urethral catheter. (**A**–**C**) Biofilm formed under the effects of acetaminophen displayed large colonization areas and compact masses bacteria in prominent islets. (**D**–**F**) Biofilm without treatment showed more empty zones with free bacterial masses. Bar 100 µm.

Analysis of the scanning electron microscope images (Figure 7) shows that the biofilm which formed under acetaminophen conditions displayed large colonization areas that were made up of compact masses of bacteria in prominent islets in comparison to those cultures without this drug.

## 4. Discussion

As mentioned before, the WHO has made an urgent call to search for new antibiotics and new treatments to control many pathogenic bacteria, including *K. pneumoniae*. This bacterium, along with *Escherichia coli*, are pathogens commonly found in urinary infections as well as forming biofilms on urethral catheters [16]. There is a significant number of strains of these bacteria resistant to first line antibiotics such as gentamicin and ciprofloxacin [17]. However, in addition to the alternatives proposed by WHO, it is also necessary to determine the biochemical mechanisms involved in the pathogenicity, virulence, and resistance of these bacteria, especially *quorum sensing*. In fact, up to the present time, there have been no published reports on the influence of drugs on the formation of these biofilms. Investigations into these mechanisms could help explain recurrent infections in patients undergoing surgical procedures.

Of the 22 drugs assayed, 11 were found capable of increasing biofilm formation and modifying *K. pneumoniae* resistance, including gentamicin, tetracycline, amikacin, norfloxacin, loratadine, captopril, verapamil, dexamethasone, and progesterone. The effects of progesterone, in addition to that of acetaminophen and hydrochlorothiazide, stand out among them. 

Progesterone displayed the greatest ability in the induction of biofilm formation (65%) but had a smaller effect on *K. pneumoniae* ATCC 13884 bacterial population growth (11%). Similarly, hydrochlorothiazide, a diuretic widely used in combination with antihypertensive drugs, had a great effect on the induction of biofilm formation (51%) and a smaller effect on bacterial growth (17%). Acetaminophen caused several changes in *K. pneumoniae* ATCC 13884 cultures related to *quorum sensing*, such as inducing the synthesis of the *N*-hexanoyl-homoserine lactone (C6-AHL) autoinducer by up 22%, increasing bacterial populations by 27%, inducing biofilm formation by 49%, and forming a more gentamicin-resistant biofilm. Furthermore, it increased the colonization area of the urethral catheter. Non-modified drugs are mainly excreted in the feces and urine at concentrations of approximately 0.09–0.6 µg/mL [18]. Therefore, intact drugs at low concentrations in urine might be enough to activate the virulence and resistance mechanisms of *K. pneumoniae* ATCC 13884. 

The hypocholesterolemic lovastatin did not cause modifications in bacterial growth or biofilm formation at concentrations of 0.024–3.1 µg/mL; however, previous studies showed that statins are able to inhibit *K. pneumoniae* growth at concentrations far higher than plasmatic ones [19]. On the other hand, metformin showed no effects on bacterial growth, but decreased biofilm formation by 21%, similar to ciprofloxacin, levofloxacin, and nitrofurantoin.

The other drugs assayed did not change the previously mentioned effects, although it has been reported that ibuprofen and diclofenac at plasmatic concentrations of 1.0 and 20 µg/mL, respectively, inhibit the formation of biofilms on abiotic surfaces [20]. 

Previously, it was established that the use of a catheter is a risk factor that increases the possibility of acquiring *K. pneumoniae* infections [21], as bacterial adherence and proliferation on abiotic surfaces causes persistent hospital infections. For instance, nearly 70% of hospitalized patients who have a urethral catheter for several days develop urinary infections due to the formation of biofilms on these devices [16]. In this paper, the effects of acetaminophen in the induction of bacterial colonization in catheters and changes in the architecture of the biofilm have been demonstrated. All of these factors favor microbial resistance to antibiotics, and, therefore, recurrence of infections.

All these results demonstrate the role of some drugs in modulating the biochemical behavior of *K. pneumoniae*. A complete understanding of the mechanism of action of these drugs in *quorum sensing* could generate new therapeutic alternatives, such as avoiding their use in high-risk patients. Furthermore, new specific inhibitors targeting this process could be designed; these working alongside current antibiotics could have the ability to restore the original power of the antibiotics through the inhibition of biofilm formation. Moreover, some bacterial development inhibitors by modulation of *quorum sensing* have been classified as anti-pathogenic [22,23] and anti-virulent [24] instead of antibiotic.

Finally, in light of the above, *quorum sensing* cannot be evaluated based only on the effect of one parameter of microbial biochemistry or by the modulation of violacein production in the biosensor *C. violaceum*, as is usually described in the literature, since microbial behavior is highly specific to each type of bacteria and autoinducer and their biochemical processes. 

## 5. Conclusions

In summary, drugs such as acetaminophen, hydrochlorothiazide, and progesterone are modulators of *quorum sensing* in *K. pneumoniae* ATCC 13884 and could be involved in bacterial resistance through the induction of a more resistant biofilm, impermeable to antibiotics. Finally, the same methodology could be used to explore the effects of drugs on other microorganisms such as *E. coli*. Similarly, it could be used to analyze the role of other molecules involved in other human activities, including foods and cosmetics, which should be considered as potential inducing agents of microbial resistance.

## Figures and Tables

**Table 1:** Plasma concentration of each drug and its pharmacological application.

Pharmacology Activity	Drug	Plasma Concentration (µg /mL)
Analgesic, Antipyretic/Anti-Inflammatory	Acetaminophen	10.0–20.0
	Diclofenac	0.015
Antibiotic	Amikacin	10.0–25.0
	Gentamicin	10.0–5.0
	Ciprofloxacin	1.6–2.9
	Levofloxacin	5.7–0.5
	Nitrofurantoin	30.0–250.0
	Norfloxacin	2.5–1.5
	Tetracycline	1.0–2.5
Anticonvulsant	Carbamazepine	10.0
Antihistamine	Cetirizine	0.025–1.0
	Loratadine	0.0025–0.1
Antihyperglycemic	Metformin	2.0
Antihypertensive	Captopril	0.47
	Propranolol	0.05–0.1
	Verapamil	0.0005–0.002
	Enalapril	0.055
Anti-Inflammatory	Dexamethasone	0.002–0.01
Bile Disorders	Cholic Acid	Up to 1.0
Cholesterol-Lowering	Lovastatin	0.022–0.036
	Hydrochlorothiazide	0.03
Hormonal Treatment	Progesterone	Up to 0.5

**Table 2:** Effect of some antibiotics on bacterial growth and biofilm formation.

Antibiotic	Concentration Range (SubMIC *) (ng/mL)	Maximum Induction of Bacterial Growth (%)	Maximum Induction of Biofilm (%)
Gentamicin	500.0–3.9	31.0	25.0
Tetracycline	170.0–1.3	31.0	46.0
Amikacin	500.0–3.9	No effect	56.0
Norfloxacin	12.0–0.09	No effect	50.0
Nitrofurantoin	12.0–0.09	No effect	−15.0 **
Levofloxacin	12.0–0.09	No effect	−15.0 **
Ciprofloxacin	1.02–0.09	No effect	−35.0 **

* SubMIC, Subminimal inhibitory concentration. ** Negative values mean biofilm inhibition effect.

**Table 3:** Effect of some drugs on bacterial growth and biofilm formation.

Drug	Concentration Range, SubMIC (µg /mL)	Maximum Induction of Bacterial Growth (%)	Maximum Induction of Biofilm (%)
Cetirizine	1–0.008	No effect	No effect
Loratadine	0.050–0.004	No effect	14.0
Lovastatin	3.1–0.024	No effect	No effect
Hydrochlorothiazide	0.050–0.004	17.0	51.0
Captopril	0.5–0.0039	7.0	24.0
Verapamil	0.5–0.0039	21.0	10.0
Propranolol	0.5–0.0039	No effect	No effect
Enalapril	0.05–0.00040	15.0	No effect
Diclofenac	0.5–0.0039	15.0	No effect
Acetaminophen	5.0–0.039	27.0	49.0
Carbamazepine	5.0–0.039	22.0	No effect
Metformin	0.5–0.0039	No effect	−21.0 *
Dexamethasone	0.5–0.0039	11.0	14.0
Progesterone	0.5–0.0039	11.0	65.0
Cholic Acid	0.5–0.0039	21.0	No effect

* Negative values mean inhibition of biofilm formation.

**Table 4:** Concentration of C6-AHL in *K. pneumoniae* ATCC 13884 cultures under the effect of biofilm-inducing drugs. Final incubation time 2 h at 37 °C.

Drug	Concentration (µg/mL)	Violacein Abs ** ± SD	Equivalent Concentration to C6-AHL ± SD (µg/mL)
Control	0.0	0.149 ± 0.030	0.259 ± 0.111
Acetaminophen *	0.6	0.165 ± 0.020	0.362 ± 0.064
Hydrochlorothiazide	0.025	0.154 ± 0.020	0.298 ± 0.065
Progesterone	0.1	0.144 ± 0.024	0.275 ± 0.097

* Statistically significant difference in respect to the control (*p* < 0.05, *p* value = 0.0084) (n = 9). ** Absorbance, arbitrary units.
